# Phenotypic Expansion in Nasu-Hakola Disease: Immunological Findings in Three Patients and Proposal of a Unifying Pathogenic Hypothesis

**DOI:** 10.3389/fimmu.2019.01685

**Published:** 2019-07-23

**Authors:** Edoardo Errichiello, Efthimios Dardiotis, Fiorenza Mannino, Juha Paloneva, Teresa Mattina, Orsetta Zuffardi

**Affiliations:** ^1^Unit of Medical Genetics, Department of Molecular Medicine, University of Pavia, Pavia, Italy; ^2^Department of Neurology, University Hospital of Larissa, University of Thessaly, Larissa, Greece; ^3^Department of Biomedical and Biotechnological Sciences, University of Catania, Catania, Italy; ^4^Department of Surgery, Central Finland Hospital, Jyväskylä, Finland; ^5^University of Eastern Finland, Kuopio, Finland

**Keywords:** Nasu-Hakola disease (NHD), *TREM2*, *TYROBP*, NK cells, autoantibodies, microglia, neurodegeneration, bone cysts

## Abstract

Nasu-Hakola disease (NHD) is a rare autosomal recessive disorder characterized by progressive presenile dementia and bone cysts, caused by variants in either *TYROBP* or *TREM2*. Despite the well-researched role of TREM2 and TYROBP/DAP12 in immunity, immunological phenotypes have never been reported in NHD patients. We initially diagnosed an Italian patient, using whole exome sequencing, with classical NHD clinical sequelae who additionally showed a decrease in NK cells and autoimmunity features underlined by the presence of autoantibodies. Based on this finding, we retrospectively explored the immunophenotype in another two NHD patients, in whom a low NK cell count and positive autoantibody serology were recorded. Accordingly, *Trem2*^−/−^ mice show abnormal levels of circulating proinflammatory cytokines and the dysfunction of immune cells, whereas knockout mice for *Tyrobp*, encoding the adapter for TREM2, exhibit increased levels of autoantibodies and defective NK cell activity. Our findings tend to redefine NHD as a multisystem “immunological” disease, considering that osteoclasts are derived from the fusion of mononuclear myeloid precursors, whereas neurological anomalies in NHD are directly caused by microglia dysfunction.

## Background

Nasu-Hakola disease (NHD; OMIM#221770 and #618193), also referred to as polycystic lipomembranous osteodysplasia with sclerosing leukoencephalopathy (PLOSL), is a rare autosomal recessive disease characterized by progressive presenile dementia and bone cysts (1). NHD is caused by loss-of-function variants in *TREM2* and *TYROBP/DAP12* (2, 3), which encode different proteins of the same receptor signaling complex, involved in the activation of the immune response. Furthermore, heterozygous variants in both genes have been involved in neurodegenerative disorders, such as frontotemporal dementia, Alzheimer's and Parkinson's diseases, and more recently, multiple system atrophy (4–6).

TREM2 (Triggering receptor expressed on myeloid cells 2) is a receptor of the immunoglobulin superfamily expressed on innate immune cells in different tissues, including the central nervous system, where it exerts a major role in regulating microglia, the sensor of neurodegeneration (7). Furthermore, TREM2 is involved in the differentiation of myeloid progenitors toward mature monocyte-derived dendritic cells (DCs), osteoclasts, and the microglia, which may be considered as the key players of NHD pathogenesis. Similarly, TYROBP/DAP12, the intracellular adaptor of TREM2, has been recognized as a key activating signal transduction element in natural killer (NK) cells (8, 9).

In this study, we provided the first evidence that NHD patients carrying biallelic variants in *TREM2* or *TYROBP* may manifest immunological abnormalities, primarily a reduction of the NK cell population.

## Clinical Data and Genetic Findings

### Case 1 (Index Case)

**Figure 1:**
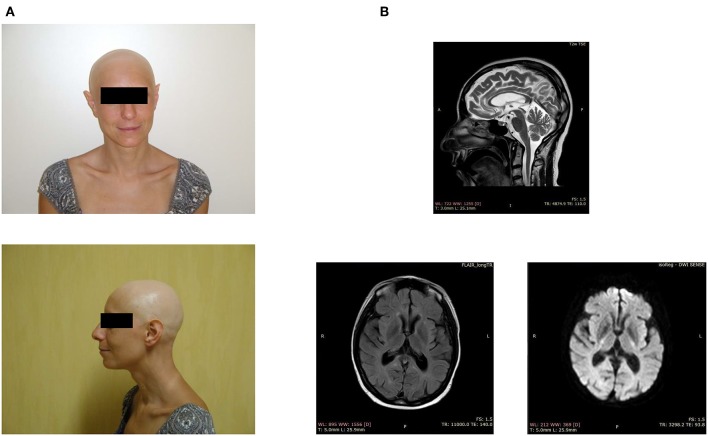
Clinical features of the index case. **(A)** Facial appearance of the patient, showing alopecia and sharp ears. **(B)** Brain MRI demonstrates periventricular white matter hyperintensities, ventriculomegaly, and a thin corpus callosum.

The index patient, a 43 year-old woman of Italian origin, is the third child of healthy parents; the eldest sister is unaffected, while the second-born died perinatally. The patient developed alopecia universalis at age 8 and painful swollen ankles and recurrent pathological fractures at 25 (Figure 1A). X-rays showed bone cysts and generalized osteopenia (T-score −1.9); abdominal ultrasound imaging identified a mild hepatomegaly with hyperechoic hepatic hemangiomas, and an inhomogeneous uterus texture with cystic areas. A bone biopsy revealed the presence of fibro-adipose tissue surrounded by hemorrhagic foci.

When the patient was around 40 years of age, we noticed the first neurological symptoms, manifesting as severe depression. The magnetic resonance imaging showed periventricular white matter hyperintensities, mild ventriculomegaly, and dilation of the subarachnoid spaces, small lacunar infarcts, a thin corpus callosum, and basal ganglia calcification (Figure 1B). The electroencephalogram, performed after the appearance of generalized seizures, revealed the absence of a normal alpha rhythm but persistent diffuse theta activity. During the next 3 years, the patient showed progressive dementia with motor disability, worsening gait impairment, sleep disturbances, spatial, and temporal disorientation, and marked memory loss, leading finally to a vegetative state.

**Figure 2:**
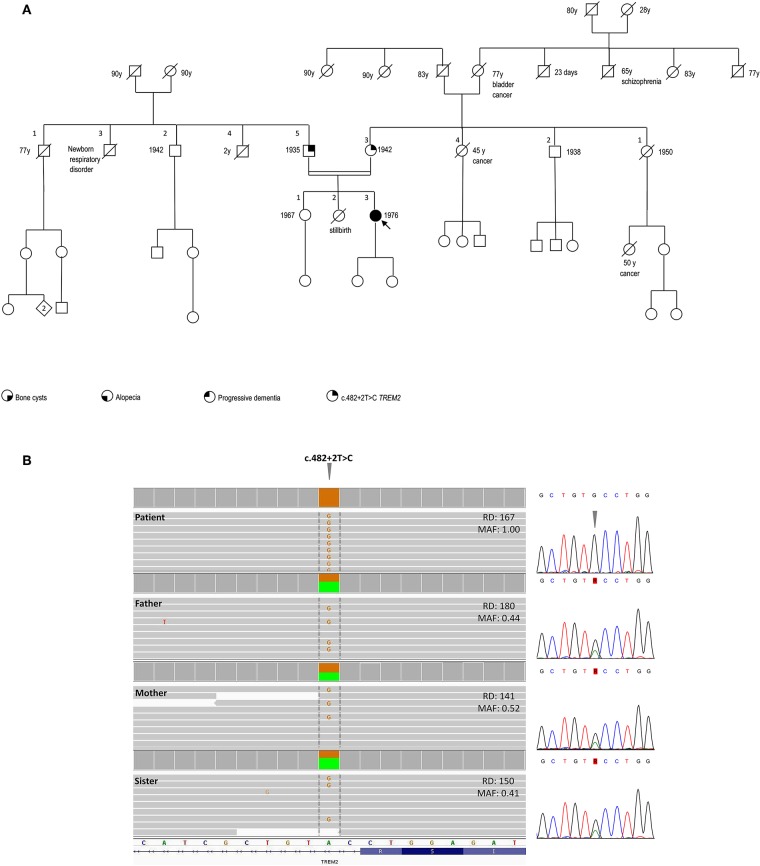
Pedigree and genetic findings of the index case. **(A)** Pedigree of the family and recorded clinical phenotypes. **(B)** IGV (Integrative Genomics View) visualization (right panel) of the chr6:41,127,528A>G (GRCh37/hg19) splice-site variant c.482+2T>C in patient (homozygous) and relatives (heterozygous). The variant, disrupting the donor splice site of *TREM2* intron 3, is annotated in both ClinVar (#RCV000050138.1) and HGMD (#CS022111) as a disease-causing/likely pathogenic and has an extremely rare allele frequency in population databases (gnomAD: 0.0008126%; ExAC: 0.00165%), with only two reported heterozygous individuals in the European Non-Finnish population and no homozygotes. The variant is included in one of the numerous regions of homozygosity (chr6:24,747,411-42,365,687). Read depths (DP) and Mutant Allele Fraction (MAF) of the variant are shown for each member of the family. Sanger sequencing confirmation of the *TREM2* variant is shown in the left panel.

The family history was negative for bone cysts, alopecia, dementia, as well as the manifestation of a dysfunctional immune system (Figure 2A).

SNP-array unveiled multiple regions of homozygosity (ROHs) in the patient's DNA likely due to the unawareness of parental consanguinity (inbreeding coefficient: 1/64, i.e., second cousins, Supplemental Table 1). Whole-exome sequencing (WES) was performed on DNA samples isolated from the peripheral blood of the patient, her parents, and the healthy sister. Libraries were generated using a commercial target enrichment kit (SureSelect Human All Exome V7, Agilent Technologies, Santa Clara, CA), and sequenced on the HiSeq 2500 platform (paired-end 2 × 100 bp; Illumina, San Diego, CA, USA), as we previously reported (10), obtaining a ~90X mean depth of coverage after mapping and removal of duplicated reads. WES identified a *TREM2* homozygous splice-donor variant (NM_018965.3:c.482+2T>C) in the proband, falling in one of the detected ROHs and affecting the splicing of exon 3, as previously demonstrated (11). This variant was at the heterozygous state in the healthy parents and sister (Figure 2B).

### Case 2

Case 2 is a 34 year-old female patient of Greek descent, originally reported by Dardiotis et al. (12). She had recurrent pathological fractures from the age of 28 and progressive cognitive and behavioral frontotemporal-like symptoms from the age of 30. At the age of 33, the patient showed a Mini-Mental State Examination (MMSE) score of 14/30, and a Montreal Cognitive Assessment (MoCA) score of 8/30. Computer tomography (CT) scan revealed cerebellar atrophy, diffuse low-density areas of the brain white matter, and subependymal and basal ganglia calcifications. Brain MRI showed hyperintensities of the white matter, brain atrophy, and a thin corpus callosum. Her condition worsened gradually, with gait instability, urinary incontinence, and memory loss. She was unable to stand and walk from the age of 37 and she underwent tracheostomy and gastrostomy at the age of 42.

The patient harbored the homozygous missense c.244G>T (p.Trp50Cys) variant in the exon 2 of *TREM2*.

### Case 3

Case 3 is a Finnish patient, originally reported by Paloneva et al. (8). The patient showed typical neurological findings and clinical evolution of NHD, including bone cysts and recurrent fractures of the ankles and wrists. Although the complete clinical history of the patient is no longer available, obvious clinical autoimmunity signs were not reported in the clinical records.

The patient carried a homozygous deletion, encompassing exons 1–4 of *TYROBP*.

## NK Cells Count and Autoantibody Screening

Flow cytometry analysis in the index case revealed low CD16+56+ NK cell counts (48 cells/μl, n.v. 90–590; 3%, n.v. 5–27%). Autoimmune screening was positive for anti-nuclear (ANA; 1:80), anti-smooth muscle (ASMA; 1:160), anti-parathyroid (1:80), and anti-Saccharomyces cerevisiae (ASCA; 1:80) autoantibodies.

Based on these results, we extended the immunological analysis to a previously diagnosed 34 year-old Greek patient harboring a homozygous missense variant in *TREM2* (case 2), in which we detected a low number of NK cells (94 cells/μl; 4.3%) as well as positive anti-parietal cell antibody (APCA; 1:320). No other autoantibodies were detected in cases 1 and 2.

In addition, clinical data of the Finnish patient (case 3) with a homozygous deletion encompassing exons 1–4 of *TYROBP*, demonstrated a low NK cell count (3.5%).

No impairment of any other cell population was detected in the three patients.

## Discussion

TREM2 and its binding partner TYROBP form an immunoreceptor signaling complex that orchestrates the differentiation of myeloid progenitors toward mature monocyte-derived dendritic cells (DCs), osteoclasts, microglia, and possibly a subset of oligodendrocytes originating in the myeloid lineage (13, 14). Curiously, *TREM2* is located on 6p21.1, contiguous to other TREM family members—TREM and TREM-like receptors (*TREM1, TREML1, TREML2, TREML3, TREML4*) expressed on myeloid cells—and relatively close (~8 Mb) to the *HLA* gene complex, thus configuring interacting genomic clusters on chromosome 6p involved in immune regulation that may have originated during evolution.

Despite the well-researched role of TREM2 and TYROBP in innate and adaptive immunity, immunological phenotypes have never been described in NHD patients, although we cannot completely rule out that subtle immunological impairments have not been sought.

The careful immunological assessment of our index patient revealed autoimmunity signs, such as persistent autoantibodies, alopecia, malabsorption, joint pain, and muscle cramps. Our findings provide the first evidence of the role of TREM2 and TYROBP in the negative regulation of inflammatory responses in patients. Although we cannot completely rule the influence of additional genetic factors on autoimmune pre-disposition out, we excluded pathogenic variants in autoimmunity-associated loci or any other gene prioritized for immune system in the index case, in which the entire exome was analyzed.

Although further studies are needed, our preliminary findings are supported by multiple lines of evidence. First, *Trem2* homozygous mutant mice show abnormal levels of circulating proinflammatory cytokines (IL-6, TNF, IFN) and dysfunction of the immune cells, osteoclasts, and microglia, as reported in the Mouse Genome Database (http://www.informatics.jax.org/). Similarly, knockout mice for *Tyrobp*, the other gene commonly associated with NHD encoding the adapter protein for TREM2, exhibits increased autoantibodies (e.g., anti-double stranded DNA) and IgG2a/3 levels, and defects of NK and T cells' regulatory function. Recently, Qu et al. (15) found an increased production of pro-inflammatory cytokines (IL-1β, IL-18, and CXCL2) and overactivation of the NLRP3 inflammasome pathway in *Trem2*^−/−^ mice.

Second, TREM2/TYROBP interact with plenty of other autoimmune-related proteins and pathways: Syk/ZAP70 protein tyrosine kinases (9), associated with autosomal recessive infantile-onset multisystem autoimmune disease-2 (OMIM #17006); LCP2/SLP-76, regulating proinflammatory cytokines, and autoantibodies production (16); PLXNA1 and SEMA6D, which modulate T-cell activation and DC maturation (17); and MIF, implicated in numerous autoimmune inflammatory disorders (18). Interestingly, *MIF* overexpression has been found in the serum and lesioned skin of patients with alopecia (19), a symptom also observed in the index patient. Furthermore, in a proteomic analysis of lymphoblastoid cells from *TREM2*-NHD patients (20), the authors found differentially expressed proteins consisting in autoantigens for α-enolase/ENO1, as reported in rheumatoid arthritis, Hashimoto's encephalopathy, and Behçet's disease, as well as tuners of immunogenic/tolerogenic balance, such as VDAC (Voltage-dependent anion-selective channel protein), which modulates thymocyte survival.

Third and more importantly, we confirmed a decrease of the NK cell population and positive autoantibody serology/autoimmunity features in additional NHD patients. Interestingly, the 34 year-old Greek female patient with *TREM2*-associated NHD in this study, has been proven to carry a reduced number of NK cells as well as positive autoantibody serology, specifically APCA. Nevertheless, the patient does not currently show manifesting signs of autoimmune atrophic gastritis nor pernicious anemia (or type 1 diabetes/autoimmune thyroiditis), although it might be considered that APCA-related manifestations may develop over many years in APCA-positive symptomless patients (21). Curiously, phagocytosis and respiratory burst of the peripheral blood cells of the patient were not significantly impaired compared to the parents and healthy controls (12). However, our current immunological findings suggest that TREM2/TYROBP impairment may rather be reflected on the specific NK cell subpopulation.

It is worth noting that regulatory NK cells are crucial for preventing autoimmune and inflammatory diseases, although the underlying mechanism(s) and the contribution of NK education remain largely to be investigated (22). Remarkably, selective NK cell deficiency is extremely rare in humans (23). To date, a few patients have been reported to carry variants in one of the following genes: *GATA2* (24), *MCM4* (25, 26), *RTEL1* (27), *GINS1* (28), *IRF8* (29), and *FCGR3A* (30). Frequently, these patients show extra-immune features—such as growth retardation, microcephaly, and adrenal insufficiency in individuals with homozygous variants in the *MCM4* gene—that did not overlap with our patients' phenotypes. Furthermore, we excluded pathogenic variants in all genes associated with the aforementioned six monogenic disorders in our index case for which the entire exome was sequenced. Therefore, we speculate that both selective NK cell deficiency and associated autoimmunological signs we documented in our patients are not coincidental events in NHD.

**Figure 3:**
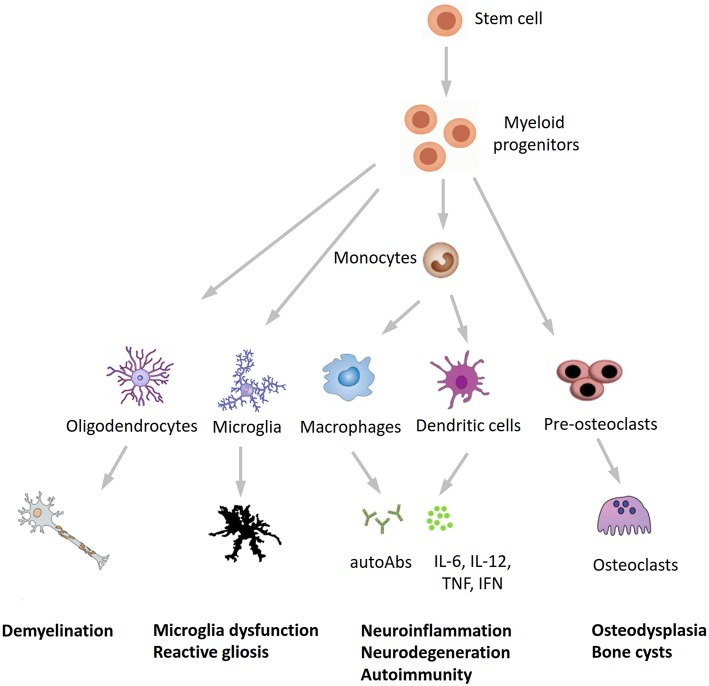
A novel hypothesis for the pathogenesis of Nasu-Hakola disease. *TREM2/TYROBP*, encoding different proteins of the same receptor signaling complex involved in the activation of the immune response, are expressed by osteoclasts, microglia, oligodendrocytes, dendritic cells, macrophages, and granulocytes. On the immune system side, the inactivation of *TREM2* or *TYROBP* may lead to the overproduction of autoantibodies and pro-inflammatory cytokines by macrophages and dendritic cells, a process which is normally restrained by the physiological presence of TREM2 and TYROBP. Finally, the inactivation of TREM2 and TYROBP is reflected in the dysfunction of different bodily systems, including bones, CNS, and (auto)immunity, all sharing a common myeloid progenitor and pointing to Nasu-Hakola disease as a “myeloid cell disease.” autoAbs, autoantibodies.

In conclusion, our findings expand the phenotypic spectrum of NHD and further underline the tight interplay between the immune and central nervous systems in neurodegeneration. Recent studies found detectable TREM2 expression in whole blood cells, with higher levels in patients with Alzheimer's disease compared to healthy subjects (31, 32), whereas Galimberti et al. (33) recently identified a specific inflammatory expression profile in peripheral blood mononuclear cells from three NHD patients carrying the pathogenic p.Gln33X variant in *TREM2*, reinforcing our hypothesis that inflammation may play a role in the pathogenesis of NHD. We hypothesize that NHD could be redefined as a multisystem “immunological” disease, considering that osteoclasts are derived from the fusion of mononuclear myeloid precursors, whereas neurological anomalies in NHD can be caused by microglia dysfunction (“disease-associated microglia”) (34) and reactive gliosis (Figure 3). Anti-inflammatory drugs or repositioning/repurposing of myeloid-specific compounds may therefore, represent a therapeutic option for modulating early stages of NHD pathogenesis, thus possibly preventing the dramatic (and often lethal) progressive neurodegeneration that characterizes patients with NHD. Whether our index patient had benefited from a potential snRNA-based approach aiming to correct mis-splicing of TREM2-mutant transcript, as recently suggested (35), remains a promising speculative hypothesis.

## Data Availability

All datasets analyzed for this study are included in the manuscript and the Supplementary Files.

## Ethics Statement

The study was conducted in accordance with the Declaration of Helsinki and national guidelines. Written informed consent was obtained from the patient's guardian for the publication of this case report and any potentially identifying images/information.

## Author Contributions

EE designed and conceptualized the study, analyzed the NGS data, made genotype-phenotype correlations, and drafted the manuscript. ED, FM, JP, and TM collected the clinical data. ED, JP, and TM helped in drafting the manuscript. OZ revised the manuscript for intellectual content and funded the study. All authors read and approved the final manuscript.

### Conflict of Interest Statement

The authors declare that the research was conducted in the absence of any commercial or financial relationships that could be construed as a potential conflict of interest.

## Supplementary Material

Supplemental Table 1 Regions of homozygosity (ROHs) in the index patient's genome.
