# Supplementary material for: Phenotypic Expansion in Nasu-Hakola Disease: Immunological Findings in Three Patients and Proposal of a Unifying Pathogenic Hypothesis
Source: Front Immunol. 2019 Jul 23;10:1685. doi: 10.3389/fimmu.2019.01685 (PMC6664049; doi:10.3389/fimmu.2019.01685)
Supplement: Supplemental Table 1 — Regions of homozygosity (ROHs) in the index patient's genome. [file Table_1.DOCX]

**Supplemental Table 1. Regions of homozygosity (ROHs) in the index patient’s genome**

| ROH | Chromosome coordinates (GRCh37/hg19) | Protein-coding genes | Length (bp) |
| --- | --- | --- | --- |
| #1 | chr6:24,747,411-42,365,687 | 365^a^ | 17,618,277 |
| #2 | chr7:54,256,062-75,806,299 | 102 | 21,550,238 |
| #3 | chr17:72,083-14,679,776 | 245 | 14,607,694 |
| Coefficient of inbreeding (*F*): 1/64^b^; Total ROH: 53.78 Mb | | | |

^a^ including *TREM2* (chr6:41,126,244-41,130,924)

^b^ corresponding to second cousins’ relationship
